# Recently Confirmed Apoptosis-Inducing Lead Compounds Isolated from Marine Sponge of Potential Relevance in Cancer Treatment

**DOI:** 10.3390/md9091580

**Published:** 2011-09-20

**Authors:** Magbubah Essack, Vladimir B. Bajic, John A.C. Archer

**Affiliations:** Computational Bioscience Research Center (CBRC), King Abdullah University of Science and Technology (KAUST), Thuwal, Jeddah 23955-6900, Kingdom of Saudi Arabia; E-Mails: magbubah.essack@kaust.edu.sa (M.E.); vladimir.bajic@kaust.edu.sa (V.B.B.)

**Keywords:** marine sponge, apoptosis, cancer treatment, targeted cancer therapy, anticancer

## Abstract

Despite intense efforts to develop non-cytotoxic anticancer treatments, effective agents are still not available. Therefore, novel apoptosis-inducing drug leads that may be developed into effective targeted cancer therapies are of interest to the cancer research community. Targeted cancer therapies affect specific aberrant apoptotic pathways that characterize different cancer types and, for this reason, it is a more desirable type of therapy than chemotherapy or radiotherapy, as it is less harmful to normal cells. In this regard, marine sponge derived metabolites that induce apoptosis continue to be a promising source of new drug leads for cancer treatments. A PubMed query from 01/01/2005 to 31/01/2011 combined with hand-curation of the retrieved articles allowed for the identification of 39 recently confirmed apoptosis-inducing anticancer lead compounds isolated from the marine sponge that are selectively discussed in this review.

## 1. Introduction

Conventional anticancer treatment such as ionizing radiation, hyperthermia, alkylating agents, DNA topoisomerase inhibitors, and platinum compounds induce DNA damage indiscriminately, thus killing the normal cell and the rapidly proliferating cancer cell. Since these drugs are not specifically selective for cancer cells, cancer patients suffer from adverse side effects [1]. These cytotoxic anticancer treatments are being supplemented by targeted therapies that recognize specific targets for cancer cell to improve efficacy of treatment and reduce side effects. Targeted therapies include apoptosis-inducers, angiogenesis inhibitors, signal-transduction inhibitors, monoclonal antibodies and gene therapy [2]. Apoptosis-inducers in particular can be used to induce cell death via the genes and proteins that control apoptosis, since tumor-specific alterations in apoptotic programs provide opportunities to target cell death in a selective manner [3]. Using these genes and proteins as potential drug targets, the apoptosis-inducers that directly induce apoptosis may provide less opportunity for acquired drug resistance, decrease mutagenesis and reduce toxicity. It is for these reasons that we have a general interest in identifying compounds that possess apoptosis-inducing capabilities. In the last decade numerous apoptosis-inducers have been isolated from marine sponges.

Marine sponges, Phylum Porifera, are the most primitive of the multicellular organisms comprising approximately 15,000 sponge species [4]. Sponges are primarily filter feeders as most rely on water constantly circulating through their porous bodies from which they filter out microorganisms and organic particles as a food source, others rely on endosymbionts to produce food and oxygen [5], while others have also been identified as carnivores that feed mainly on small crustaceans [6]. Sponges, and/or their symbionts, produce a plethora of secondary metabolites to repel and deter predators [7,8] and to compete for space with other sessile marine organisms [9]. Sponges are acknowledged as the most versatile source of marine natural products with biomedical applications [10–12].

The first successful sponge-derived pharmaceutical drugs were the nucleosides spongothymidine and spongouridine which were isolated from *Tectitethya crypta* (formerly *Cryptotethya crypta*) [13,14]. A derivative of these nucleosides, Ara-C (also known as 1-beta-d-Arabinofuranosylcytosine or cytarabine) is documented as the first marine derived anticancer agent [15] that is currently used for the treatment of leukemia [16]. Ara-C is also screened in several clinical trials for the treatment of acute myeloid leukemia (AML) in combination with drugs such as Daunoribicin [17], Clofarabine [18] and Cladribine [19].

Despite the technological challenges in characterizing and scaling up production of bioactive compounds from marine sponges [20], several sponge-derived anticancer compounds are in preclinical/clinical trials [21–24]. Here, we review recently confirmed apoptosis-inducing anticancer lead compounds isolated from marine sponges which may have the potential to be future drug molecules. We used the National Center for Biotechnology Information (NCBI) PubMed database [25] to search for marine sponge derived anticancer compounds using the following keywords:

‘marine sponge’ AND (programmed cell death OR apoptosis OR antitumor* OR anti-tumor* OR anticancer* OR anti-cancer* OR antineoplasm* OR anti-neoplasm*)

This query was limited to articles published from 01/01/2005 to 31/01/2011, so as to include only recently confirmed apoptosis inducing lead compounds isolated from marine sponges. This yielded a total of 218 documents, curation of which allowed for the identification of 39 apoptosis-inducing lead compounds (Table 1) that have the potential to be developed as anticancer drugs.

## 2. Extensively Studied Apoptotic Pathways

### 2.1. The Death Receptor or “Extrinsic” Apoptosis Pathway of Type I and Type II

**Figure 1:**
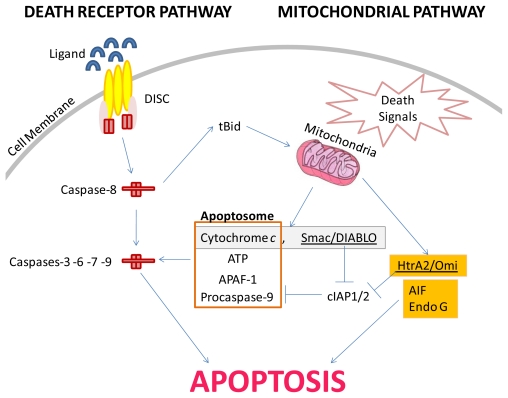
Schematic illustration of the death receptor (type I and type II) and mitochondrial pathways.

The extrinsic apoptosis signaling pathway is mediated by the activation of cell surface receptors (death receptors) that ligate with specific ligands to form a death inducing signaling complex (DISC) that transmits the apoptotic signals. Some DISC include CD95/Fas (CD95L/FasL) [54], TNFR1 (TNF) [55], DR3 (Apo3L) [56], DR4 (TRAIL-R1) [57] and DR5 (TRAIL-R2) [57]. The procaspase-8 is then sequestered by the DISC resulting in high local concentrations of zymogen. Under this condition, the low intrinsic protease activity of pro-caspase-8 is sufficient to allow various proenzyme molecules to mutually cleave and activate each other resulting in active caspase-8 [58]. Active caspase-8 then directly activates downstream effector caspase-3 resulting in the death of cells classified as type I cells (Figure 1) [59].

In type II cells, the above described signal requires amplification by the simultaneous activation of the mitochondria-dependent apoptotic pathway [59]. The mitochondria-dependent apoptotic pathway is activated when active caspase-8 additionally cleaves Bid to form the truncated Bid (tBid) which translocates to the mitochondria to induce outer membrane permeabilization whilst the inner membrane permeabilization that is promoted by the mitochondrial permeability transition pore formed across inner membranes when Ca^2+^ reaches a critical threshold [60,61]. Permeabilization of the membrane leads to the release of apoptogenic factors including cytochrome *c* [62], apoptosis-inducing factor (AIF) [63], second mitochondria-derived activator of caspases (Smac/Diablo) [64], EndoG [65] and HtrA2/Omi [66]. Cytochrome *c* and Smac/DIABLO activates the caspase dependent pathway. The cytochrome *c* disrupts the binding between Bcl-2 and pro-apoptotic protease activating factor (Apaf-1). The unbound Apaf-1 binds to procaspase-9 form the Apaf-1/caspase-9 complex that together with ATP and cytochrome *c* forms the apoptosome [67]. The apoptosome activates caspase-3 and -7 [68]. Caspase-3 successively mediated cleavage of (1) inhibitor caspase-activated deoxyribonuclease IFN (ICAD) resulting in the release and activation of the catalytic subunit CAD which is responsible for the fragmentation of the DNA into approximately 200 bp fragments [69], and (2) poly (ADP-ribose) polymerase (PARP) [70] and caspase-6 [71]; thus impeding the DNA repair mechanism, and bringing forth lamin protein degradation along with the disturbance of the fodrin and gelsolin matrix respectively [72,73]. Smac/DIABLO aids the caspases in dismantling of the cell by binding to and deactivating the inhibitor of apoptosis proteins (IAPs), thus preventing the IAPs from arresting the apoptotic process [74]. The other apoptogenic factors such as AIF and Endo G promotes DNA fragmentation in a caspase-independent manner to execute cell death [65,75]. AIF additionally induces the negatively charged phosphatidylserine to externalize on the cell membrane [76]. HtrA2/Omi is a Smac/DIABLO-like inhibitor of IAP activity that acts in a caspase-independent manner to execute cell death [66]. As a consequence of the above sequence of events, apoptotic cells display characteristic morphology changes such as cellular shrinking, chromatin condensation and margination at the nuclear periphery which ultimately results in membrane-bound apoptotic vesicles that are phagocytosed without triggering inflammatory responses that are distinct from that seen with necrotic cells [77].

### 2.2. The Mitochondrial or “Intrinsic” Apoptosis Pathway

The mitochondrial pathway can be activated by a variety of receptor-independent stimuli such as radiation, chemotherapeutics, hypoxia, ROS, Bax, p53 and viral infections [78]. Exposure to radiation or chemotherapeutics induces DNA damage that leads to the activation of p53 [79]. Activated p53 initiated the expression of (1) p21 that binds to the cyclin/kinase complex giving rise to a cell cycle restriction site in which an attempt is made to repair the DNA, and (2) GADD + PCNA that attempts to repair the DNA damage. If the DNA damage is irreparable p53 further initiated the expression of the Bax [80]. The increased levels of Bax increase the concentrations of Bax/Bax complexes (proapoptotic complex) that have been demonstrated to displace the Bcl-2/Bax complexes (anti-apoptotic complex) that compete for the same receptor site on the mitochondrial membrane [81]. The binding of the Bax/Bax complex to the mitochondrial membrane brings forth the release of the apoptogenic factors that signifies the point where the intrinsic and extrinsic pathways converge (refer to Figure 1).

## 3. Apoptosis-Inducing Lead Compounds

**Figure 2:**
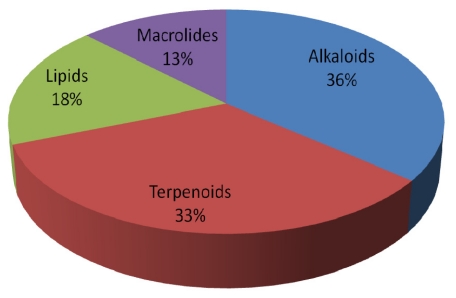
Recently confirmed apoptosis-inducing lead compounds isolated from marine sponge since the 01/01/2005, divided by putative biogenetic origin.

An overview of the 39 apoptosis-inducing lead compounds from marine sponge reported in literature since 01/01/2005, based on their putative biogenetic origin is presented in Figure 2. The compounds identified comprised 14 alkaloids, 13 terpenoids, 7 lipids and 5 macrolides. The disproportionately high frequency of alkaloids and terpenoids was also observed by Blunt *et al.* (2008) [82].

### 3.1. Alkaloids

#### 3.1.1. Renieramycin M

**Figure 3:**
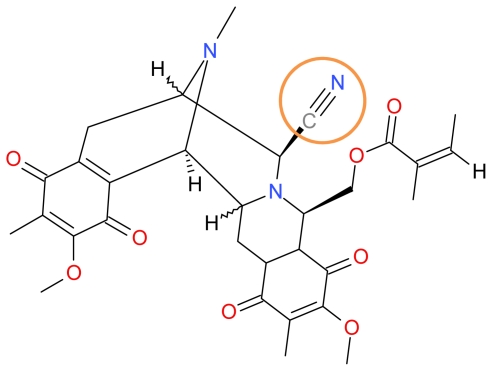
Structure of Renieramycin M.

Renieramycins are members of the tetrahydroisoquinoline family that were isolated from marine sponges belonging to genera *Reniera* [83], *Xestospongia* [84,85], *Cribrochalina* [86] and *Neopetrosia* [87]. A recent study by Halim *et al.* demonstrated that Renieramycin M (Figure 3) isolated from the *Xestospongia* species induced apoptosis via the p53-dependent pathway and inhibits the progression and metastasis of non-small lung cancer cells [32]. Other members of this family such as Renieramycin G and its analog 3-*epi*-renieramycin G, both of which possess an amide carbonyl group at the C-21 position demonstrated minimal antiproliferative activity in human colorectal cancer (HCT-116) and human lung adenocarcinoma (A549) cells owing to both compounds possessing an amide carbonyl group at the C-21 position [88]. The minimal antiproliferative activity for members of the tetrahydroisoquinoline family possessing an amide carbonyl group at the C-21 position seems consistent compared to their C-21 cyano- or carbinolamine-containing relatives [89].

#### 3.1.2. Naamidine A

**Figure 4:**
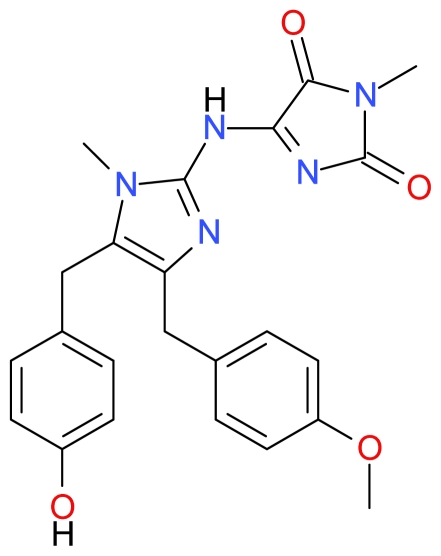
Structure of Naamidine A.

Copp *et al.* isolated Naamidine A (Figure 4) from the Fijian marine sponge *Leucetta chagosensis* and demonstrated that the compound inhibits the EGF signaling pathway and is more specific for the EGF-mediated mitogenic response than for the insulin-mediated mitogenic response [90]. In 2009, LaBarbera *et al.* demonstrated that Naamidine A induces biomarkers of apoptosis such as externalization of phosphatidylserine, disruption of the mitochondrial membrane potential, and cleavage and activation of caspases-3, -8, and -9, suggesting that the cell death induced by Naamidine A in epidermoid carcinoma (A-431) cells is a result of apoptosis rather than cytotoxicity [30]. Naamidine A was also shown to inhibit tumour xenograft growth via activation of caspase-3 suggesting apoptosis activity *in vivo*. Furthermore, Naamidine A-induced apoptosis is independent of extracellular signal-regulated kinase 1/2 and does not require functional p53 [30].

#### 3.1.3. Psammaplysene A

**Figure 5:**
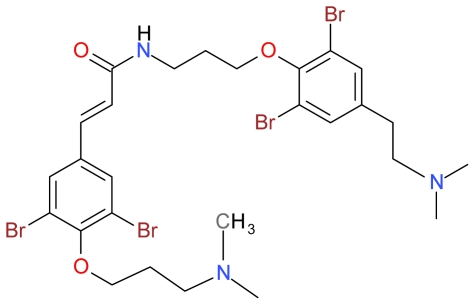
Structure of Psammaplysene A.

Schroeder *et al.* (2005) isolated Psammaplysene A and B (Figure 5) from the Indian Ocean marine sponge, *Psammaplysilla* sp. when screening for specific inhibitors of nuclear export of the transcription factor FOXO1a [91]. Schroeder *et al.* demonstrated that both Psammaplysene A and B were active inhibitors of FOXO1 nuclear export but Psammaplysene A was the more potent inhibitor [91]. Berry *et al.* further demonstrated that human endometrial cancer (Ishikawa and ECC-1) cells treated for 24 h with 1 μM Psammaplysene A induces cell death displaying morphology changes consistent with apoptosis and PARP cleavage, suggesting that the cell death induced by Psammaplysene A is a consequence of apoptosis rather than cytotoxicity. Psammaplysene A was also shown to induce the doubling of cells in the G2/M phase and FOXO1 silencing in ECC-1 cells lessens Psammaplysene A-induced apoptosis [31].

#### 3.1.4. Monanchocidin

**Figure 6:**
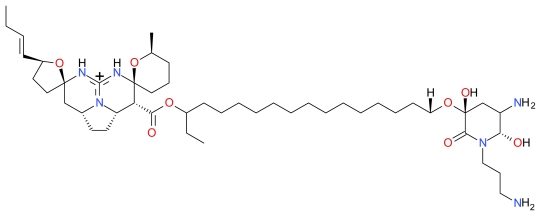
Structure of Monanchocidin.

Monanchocidin (Figure 6) is a novel polycyclic guanidine alkaloid isolated from the marine sponge *Monanchora pulchra* [29]. Guzii *et al.* demonstrated that Monanchocidin induces cell death in human monocytic leukemia (THP-1), human cervical cancer (HeLa) and mouse epidermal (JB6 Cl41) cells. Additionally, they demonstrated that THP-1 cells treated for 12 h with 1 μM Monanchocidin induces externalization of phosphatidylserine, suggesting that cell death induced by Monanchocidin may be a consequence of apoptotic as opposed to necrotic cell death [29].

#### 3.1.5. Kuanoniamines A and C

**Figure 7:**
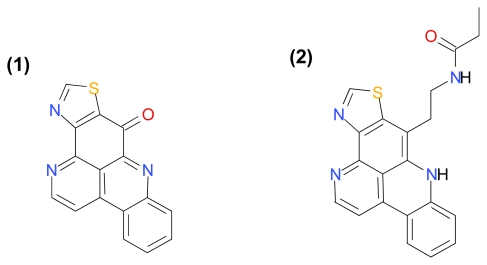
Structure of Kuanoniamines A (**1**) and C (**2**).

Kuanoniamines A and C (Figure 7), pyridoacridine alkaloids isolated from the Thai marine sponge *Oceanapia sagittaria* were shown to be growth inhibitors of breast adenocarcinoma (estrogen-dependent ER (+)) (MCF-7), breast adenocarcinoma (estrogen-independent ER (−)) (MDA-MB-231), non-small cell lung cancer (NCI-H460), diploid embryonic lung fibroblast (MRC-5), glioblastoma (SF-268) and melanoma (UACC-62) cells [27]. Kuanoniamine C was less potent but showed high selectivity toward the estrogen dependent (ER+) MCF-7 cells. It was further demonstrated that Kuanoniamine A is a more potent inhibitor of DNA synthesis than Kuanoniamine C, and Kuanoniamine A was shown to cause an extensive reduction of the MCF-7 cells in the G2/M phase. It was also demonstrated that MCF-7 cells treated for 24 h with Kuanoniamine A (0.5 and 1 μM) and Kuanoniamine C (1 and 2.5 μM) respectively, displayed DNA fragmentation consistent with apoptosis [27]. Interestingly, in 1994, MacDonald *et al.* isolated the congeners Dehydrokuanoniamine B and Kuanoniamine D from the Fijian *Cystodytes* sp. ascidian and demonstrated that these compounds inhibit TOPO II-mediated decatenation of kinetoplast DNA correlated with their cytotoxic potencies and their ability to intercalate into calf thymus DNA [92]. Taken together, Kuanoniamines A and C likely inhibit DNA synthesis via the same mechanism as Dehydrokuanoniamine B and Kuanoniamine D.

### 3.2. Terpenoids

#### 3.2.1. Rhabdastrellic Acid-A

**Figure 8:**
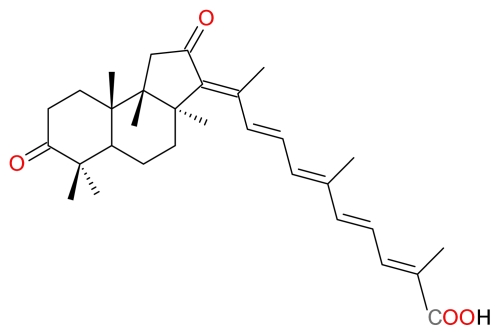
Structure of Rhabdastrellic acid-A.

In 1997, Rao *et al.* isolated Rhabdastrellic acid-A (Figure 8), a novel triterpenoid from the marine sponge *Rhabdastrella globostellata*. Ten years later, Guo *et al.* demonstrated that human promyelocytic leukemia (HL-60) cells treated for 36 h with 1 μM Rhabdastrellic acid-A induces condensation of nuclear chromatin and DNA fragmentation. Rhabdastrellic acid-A was also shown to induce other hallmarks of apoptosis such as PARP cleavage and caspase-3 activation [51]. They additionally demonstrated that mRNA levels of 44 genes, including p73, JunD, TNFAIP3 and GADD45A, were up-regulated whilst the mRNA levels of 16 genes, including MAP2K5 and IGF2R, were down-regulated [50]. Li *et al.* demonstrated that Rhabdastrellic acid-A also induces caspase-independent autophagy-associated cell death through blocking the Akt pathway in heptocellular carcinoma (Hep3B) and A549 cells [93].

Li *et al.* further isolated nine new isomalabaricane-derived natural products, Globostelletins A–I from the marine sponge *Rhabdastrella globostellata*, together with Jaspolides F, Rhabdastrellic acid-A, (−)-Stellettin E, Stellettins C and D [94]. They demonstrated that all the compounds induced inhibitory activities in A549, human gastric gland carcinoma (BGC-823), human intestinal adenocarcinoma (HCT-8) and human hepatocellular carcinoma (Bel-7402) cells and confirmed that HL-60 cells treated for 24 h with 5 μM Rhabdastrellic acid-A induces externalization of phosphatidylserine characteristic of apoptotic cell death [94].

#### 3.2.2. Stellettin A and B, and Geoditin A and B

**Figure 9:**
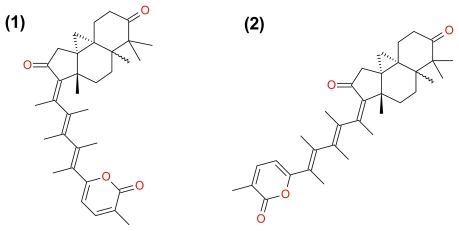

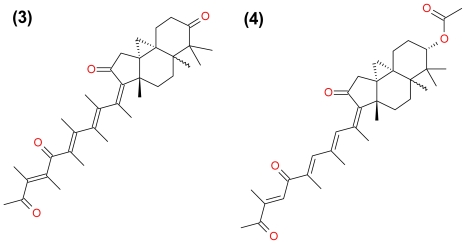
Structure of Stellettin A (**1**) and B (**2**), and Geoditin A (**3**) and B (**4**).

Zhang and Che isolated four isomalabaricane triterpenes (Stellettin A, Stellettin B, Geoditin A and Geoditin B) (Figure 9) from marine sponge *Geodia japonica* [95]. Liu *et al.* demonstrated that Stellettin A, Stellettin B, Geoditin A and Geoditin B induce cytotoxicity in HL-60 cells with Geoditin A being the most potent, followed by Stellettin A and B while Geoditin B showed the least potency. Liu *et al.* further demonstrated that HL-60 cells treated for 24 h with 3 μM of all four compounds respectively, induced dissipation of mitochondrial membrane potential, activation of caspase-3, and a decrease in levels of proliferating cell nuclear antigen (PCNA) characteristic of apoptotic cell death [46]. It was also demonstrated that apoptosis induced by Geoditin A displayed a dose-dependent increase of reactive oxygen species (ROS) and an increase in externalization of phosphotydilserine [46].

#### 3.2.3. Jaspolide B

**Figure 10:**
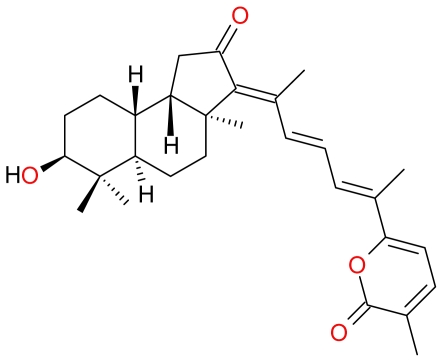
Structure of Jaspolide B.

Jaspolide B (Figure 10), a novel isomalabaricane-type triterpene was isolated from the sponge *Jaspis* sp. [50,96]. Wei *et al.* demonstrated that Bel-7402 and human hepatocellular liver carcinoma (HepG2) cells treated for 24 h with 20 μM Jaspolide B induces increased mitochondrial masses and cell membrane permeability, as well as nuclear condensation characteristic of apoptotic cell death. Jaspolide B was also shown to induce cell cycle arrest in the G_1_ phase in both Bel-7402 and HepG2 cells [50]. Moreover, Jaspolide B induced disassembly of the microtubule cytoskeleton in Bel-7402 cells, the effect of which being similar but weaker than that of the known microtubule-disassembly agent, colchicines [50].

#### 3.2.4. Smenospongine

**Figure 11:**
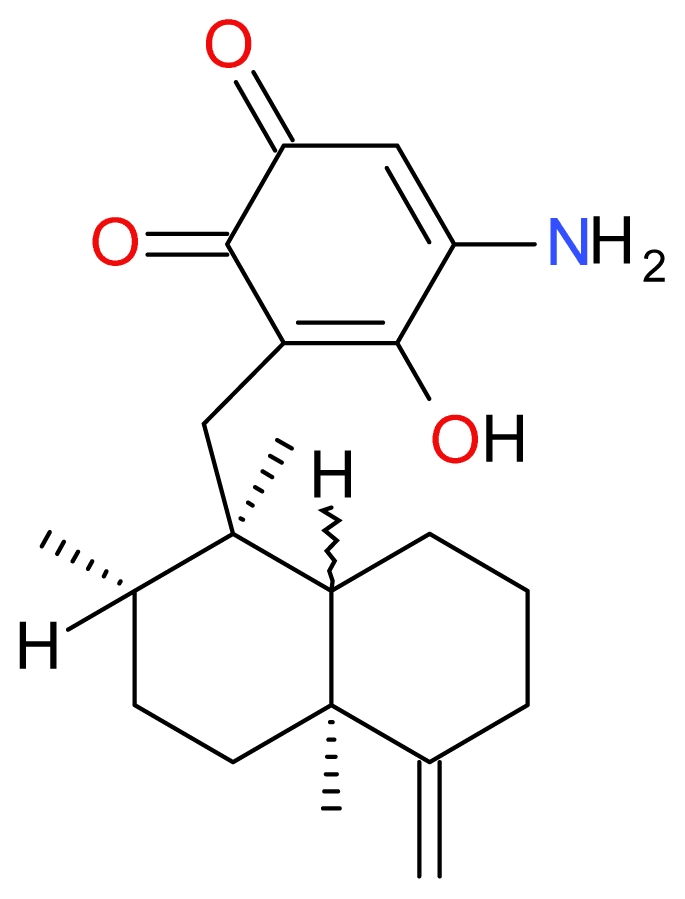
Structure of Smenospongine.

Kondracki and Guyot were the first to isolate Smenospongine (Figure 11) from the *Smenospongia* sp. as early as 1987 and demonstrated that Smenospongine induces cytotoxic and antimicrobial activity [97]. Later, Aoki *et al.* isolated Smenospongine from the Indonesian marine sponge *Dactylospongia elegans* [44,98]. It was demonstrated that Smenospongine induced the differentiation of human erythromyeloblastoid leukaemia (K562) cells into erythroblasts, cell-cycle arrest in the G1 phase and increased expression of p21 [98]. In addition, human leukemic monocyte lymphoma (U937) cells treated for 24 h with 15 μM Smenospongine induced DNA fragmentation characteristic of apoptotic cell death [53]. Kong *et al.* further demonstrated that smenospongine-induces antiproliferative and antiangiogenic activities such as endothelial migration and tube formation of human umbilical vein endothelial cells (HUVECs) [99].

#### 3.2.5. 13*E*,17*E*-Globostellatic Acid X Methyl Ester

**Figure 12:**
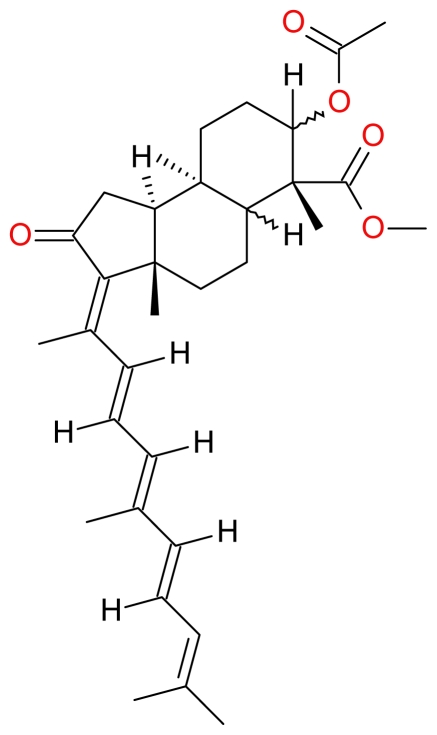
Structure of 13*E*,17*E*-globostellatic acid X methyl ester.

Aoki *et al.* isolated seven new compounds (13*Z*,17*Z*-globostellatic acid X methyl ester, 13*Z*,17*E*-globostellatic acid X methyl ester, 13*E*,17*Z*-globostellatic acid X methyl ester, and 13*E*,17*E*-globostellatic acid X methyl ester (Figure 12), Acetyljaspiferal E, Globostellatic acid F methyl ester and 13*E*-globostellatic acid B methyl ester) from the marine sponge *Rhabdastrella globostellata*, as selective anti-proliferative agents of HUVECs [44]. They demonstrated that HUVECs treated for 48 h with 1 μM 13*E*,17*E*-Globostellatic acid X methyl ester induces caspase-3 and -7 activity, suggesting that 13*E*,17*E*-globostellatic acid X methyl induced cell death may be a consequence of apoptosis [44]. Aoki *et al.* further demonstrated that 13*E*,17*E*-Globostellatic acid X methyl ester also induces antiangiogenic activities such as endothelial migration and tube formation of HUVECs [44].

### 3.3. Lipids

#### 3.3.1. Petrosterol-3,6-dione and 5α,6α-*epoxy*-Petrosterol

**Figure 13:**
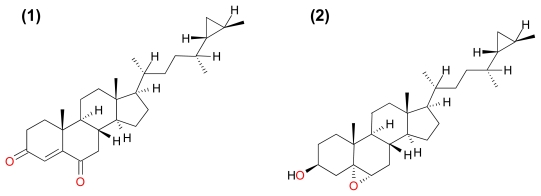
Structure of Petrosterol-3,6-dione (**1**) and 5α,6α-*epoxy*-petrosterol (**2**).

Nguyen *et al.* isolated two new C_29_ sterols, Petrosterol-3,6-dione and 5α,6α-*epoxy*-petrosterol (Figure 13) along with the known C_29_ sterol, Petrosterol from the Vietnamese marine sponge *Ianthella* sp. [35]. It was shown that all three compounds induced cell death in A549, HL-60, U937, MCF-7 and human ovarian carcinoma (SK-OV-3) cells. To further evaluate whether this cell death was necrotic or apoptotic, the HL-60 cells were treated for 24 h with IC_50_ (19.9, 21.3, and 21.5 μM) of each compound (Petrosterol-3,6-dione, 5α,6α-*epoxy*-petrosterol and Petrosterol) respectively. Each compound induced apoptotic events such as chromatin condensation and DNA fragmentation with Petrosterol being the more potent apoptosis inducer [35]. Since the compounds induced apoptotic cell death in HL-60 cells, it is expected to be able to induce apoptotic cell death in the other cells tested but which compound will be the most potent in the other cell lines and the mechanisms of cell death induced by these compounds based on cancer cell types remains unresolved.

### 3.3.2. Pachastrissamine

**Figure 14:**
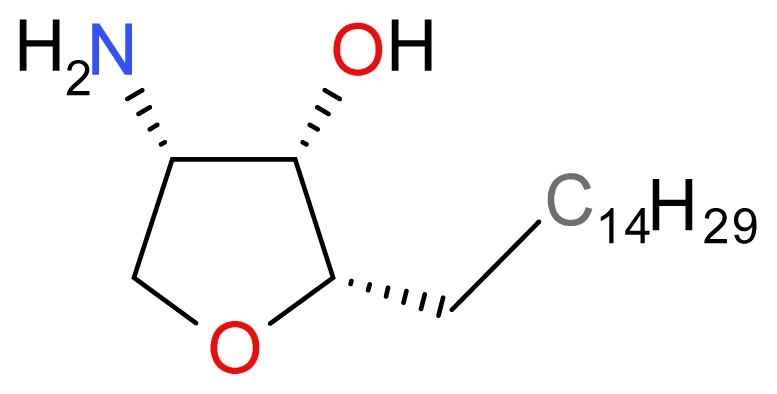
Structure of Pachastrissamine.

Kuroda *et al.* originally isolated the anhydrophytosphingosine, Pachastrissamine (also known as Jaspine B) (Figure 14) from the marine sponge *Pachatrissa sp*. and demonstrated that Pachatrissamine induced cytotoxicity in mouse lymphocytic leukemia (P388), A-549, human colon adenocarcinoma (HT-29) and human melanoma (SK-Mel-28) cells [100]. Salma *et al.* further demonstrated that B16 mouse melanoma cells induced for 6 h with 5 μM Pachastrissamine trigger hallmarks of apoptosis such as externalization of phosphatidylserine, release of cytochrome *c*, PARP cleavage and activation of caspase-3 and -9. Moreover, they demonstrated that Pachastrissamine inhibited the activity of sphingomyelin synthase (SMS), an enzyme that converts ceramide into the membrane lipid sphingomyelin. To evaluate whether this inhibitory activity played a role in the Pachastrissamine-induced cell death mechanism, they further demonstrated that cell death was enhanced in SMS1-depleted cells, whilst it was strongly inhibited in cells that stably overexpress human SMS1 [37]. Taken together, ceramide formation plays a key role in activating the mitochondrial-dependent pathway induced by Pachastrissamine.

#### 3.3.3. Rhizochalin

**Figure 15:**
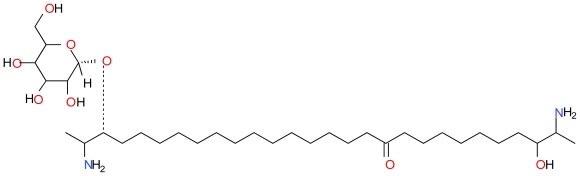
Structure of Rhizochalin.

Rhizochalin (Figure 15) is a two-headed sphingolipid isolated from the Madagascarian sponge *Rhizochalin incrustata* [101]. Jin *et al.* demonstrated that HL-60 cells induces for 24 h with 10 μM Rhizochalin display hallmarks of apoptosis such as disruption of the mitochondrial membrane potential, cleavage of PARP and activation of caspase-3, -8 and -9 in HL-60 cells [102]. The activation of caspase-8 suggests that this two-headed sphingolipid induces a death receptor pathway.

### 3.4. Macrolides

#### 3.4.1. Salarin C

**Figure 16:**
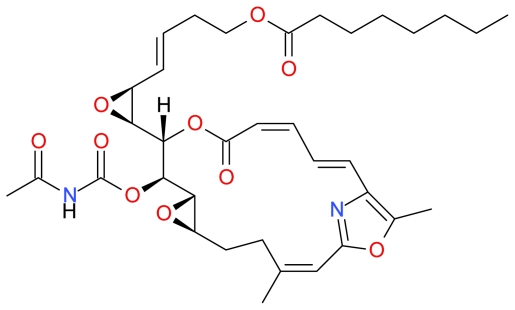
Structure of Salarin C.

Ben-Califa *et al.* isolated Salarin A–J from the marine sponge Madagascar *Fascaplysinopsis* sp. and demonstrated that Salarin C (Figure 16) is the most potent proliferation inhibitor of K562 cells [42]. It was further shown that K562 cells induced for 24 h with 0.1 μM Salarin C-displayed numerous hallmarks of apoptosis such as externalization of phosphotydilserine, cleavage of PARP and activation of caspase-3 and -9 [42].

#### 3.4.2. Spongistatin 1

**Figure 17:**
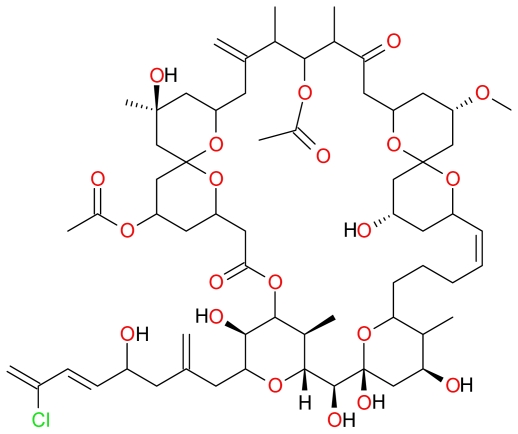
Structure of Spongistatin 1.

The macrocyclic lactone polyether Spongistatin 1 (Figure 17) was isolated as early as 1993 from the marine sponge *Spongia* sp. [103,104]. Spongistatin 1 was shown to inhibit mitosis, microtubule assembly, and the binding of vinblastine to tubulin thereby inducing cytotoxic cell death in numerous cancer cell lines [104]. Fifteen years later, Schyschka *et al.* demonstrated that Spongistatin 1-induced human acute leukemia (Jurkat T) cells displayed hallmarks of apoptosis such as mitochondrial-released of cytochrome *c*, Smac/DIABLO and Omi/HtrA2 and caspase activation [43]. The activation of caspase-9 and suppression of Spongistatin 1-induced apoptosis by overexpressed Bcl-2 and Bcl-xL indicates that Spongistatin 1-induces apoptosis through the mitochondrial pathway. Moreover, Spongistatin 1 was shown to degrade XIAPs and induce cell death in leukemic tumor cells that are known to be resistant to apoptosis induction due to the overexpression of XIAPs [43].

Schneiders *et al.* additionally demonstrated that MCF-7 cells treated for 8 h with 500 pM Spongistatin 1 displayed activation of Bim and by 16 h Bim had mediated the translocation of AIP and endo G from the mitochondria. They further used gene silencing to confirm that the Spongistatin 1-induced apoptosis is primarily via a caspase-independent signaling pathway [105] Rothmeier *et al.* further demonstrated that Spongistatin 1 is a microtubule-depolarizing compound that induces endothelial migration, tube formation, G2/M arrest, and induction of apoptosis at roughly the same inhibitory concentration (1 nM) [106]. Spongistatin 1 was demonstrated to be a more potent inhibitor of angiogenesis than Vinblastine, CA4P, and Paclitaxel, as Spongistatin 1-induced proliferation 100 pM, migration 1.0 nM, tube formation 1.0 nM, chemotaxis 1.0 nM and aortic ring sprouting 500 pM.

#### 3.4.3. Candidaspongiolide

**Figure 18:**
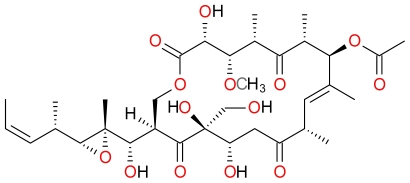
Structure of Candidaspongiolide.

Meragelman *et al.* isolated Candidaspongiolide (Figure 18) from the marine sponge *Candidaspongia* sp. and demonstrated that this compound induced potent cytotoxicity in melanoma and glioma cells [107]. Trisciuoglio *et al.* further demonstrated that HCT-116 and human glioblastoma (U251) cells treated for 24 h with 50 μM Candidaspongiolide induced hallmarks of apoptosis such as cleavage of PARP and activation of caspase-3 and -12. They further demonstrated that Candidaspongiolide induced a non-classic PKR/eIF2alpha/caspase-12-dependent apoptotic pathway and inhibited protein synthesis in HCT-116 and U251 cells [39]. While in normal fibroblasts Candidaspongiolide only inhibited protein synthesis [39].

#### 3.4.4. Irciniastatin A

**Figure 19:**
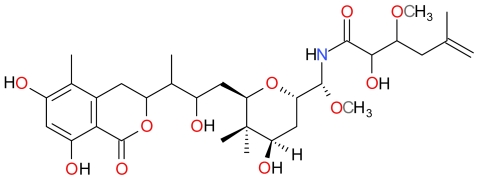
Structure of Irciniastatin A.

Irciniastatin A (also known as Psymberin) (Figure 19) was isolated by Pettit *et al.* from the marine sponge *Ircinia ramose* [108], and by Cichewicz *et al.* from the marine sponge *Psammocinia* sp. [107]. Cichewicz *et al.* demonstrated that Irciniastatin A induced cytotoxicity in human lung melanoma (MALME-3M), human skin melanoma (SK-MEL-5), p53 wild-type human melanoma (UACC-62), HCT-116 and human breast adenocarcinoma (MDA-MB-435) cells [109]. Chinen *et al.* further demonstrated that Jurkat T cells treated for 3 h with 10 nM Irciniastatin A induced hallmarks of apoptosis such as capase-8, -9 and -3, suggesting that Irciniastatin A induces a death receptor pathway [40].

## 4. Discussion

Because of the pressing need to develop non-cytotoxic anticancer treatments, novel apoptosis-inducing drug leads that have the potential to be developed into effective targeted cancer therapies are of interest to the cancer research community. In this regard, marine sponge-derived bioactive metabolites continue to be one of the most promising sources of new drug leads. The Blunt *et al.* yearly reviews of literature published for marine natural products covered 5666 novel compounds isolated from 2002 to 2008, with approximately one third of these compounds being isolated from marine sponge [82,110–115]. Many of these sponge-derived novel compounds have been screened for cytotoxic activity, but not apoptosis [116–118].

Cytotoxic activity is regarded as the first indicator in identifying anticancer drugs [119]. However, increasing evidence has shown that cell death can be induced via three different mechanisms: apoptosis (programmed cell death type I), autophagy (programmed cell death type II, or lysosomal cell death), and oncosis (programmed cell death type III, also referred to as necrosis) [120]. Cell death by autophagy is characterized by: (a) being mainly caspase-independent; (b) organelles degrading at an early stage of cell death; (c) the cytoskeleton remaining preserved until the final phase of the dying process; and (d) the formation of autophagosomes that fuse together with lysosomes which degrade the autophagosomes content [120]. Marine sponge derived cytotoxic compounds such as the aminosteroid clionamine A [121], the alkaloid xestospongin B [122] and the triterpenoid rhabdastrellic acid-A [93] were shown to induce cell death by autophagy. Oncosis is characterized by: (a) cellular swelling; and (b) cell membrane bursting that releases the cellular contents into the environment causing inflammatory cell suicide [120]. In contrast with autophagy and oncosis, apoptosis is the natural cell death mechanism that is characterized by: (a) chromatin condensation; (b) nuclear fragmentation and chromosomal DNA fragmentation (karyorrhexis); (c) alterations of the cellular membrane; (d) cellular shrinkage; and (e) the formation of vesicles that are phagocytosed by macrophages leading to non-inflammatory cell suicide [120]. The mechanisms through which lead compounds induce apoptosis remains debatable; however, cell death via apoptosis induction is widely accepted as an effective strategy for the identification of potential anticancer drugs.

The efficacy of treatment with apoptotic drugs is however limited by their toxicity to normal cells [123]. One of the recently confirmed apoptosis-inducing lead compounds isolated from marine sponges, Candidaspongiolide was shown to induce a PKR/eIF2alpha/caspase 12-dependent apoptotic pathway in HCT-116 and U251 cells, but not in normal fibroblasts [39].

The apoptotic pathway induced and the structure of the apoptosis-inducing lead compound allow for the targeting of selective cancer types. Irciniastatin A in particular, was shown to induce selective cell killing against melanoma (MALME-3M, SK-MEL-5 and UACC-62), colon (HCT-116) and breast (MDA-MB-435) cancer cells [109]. Also, the similarly structured Kuanoniamines A and C induced selective cell killing against breast (MCF-7 and MDA-MB-231), lung (NCI-H460 and MRC-5), glioblastoma (SF-268) and melanoma (UACC-62) cancer cells [27], with Kuanoniamine C inducing less potent cell death than Kuanoniamines A but displaying high selectivity toward the estrogen dependent (ER+) breast (MCF-7) cancer cells [27]. It was also shown that a caspase-dependent apoptotic pathway is induced in the HL-60 cells by the similarly structured isomalabaricane triterpenes (Stellettin A, Stellettin B, Geoditin A and Geoditin B) [46]. Apoptosis-induction varies among these four isomalabaricane triperpenes, with Geoditin A being the most effective inducer, followed by Stellettin A, Stellettin B and Geoditin B, respectively [46]. However, it remains unclear whether there is a difference in the apoptosis pathways and the potency of apoptosis induced by the isomalabaricane triperpenes, and which compound type is a more suitable candidate lead compound to be developed into an anticancer drug that targets leukemia.

Other compounds such as Rhabdastrellic acid-A has been shown to induce different types of cell death, as it was demonstrated that Rhabdastrellic acid-A induced caspase-dependent apoptosis in HL-60 cells [51], but to the contrary, induces caspase-independent autophagy-associated cell death through blocking the Akt pathway in Hep3B and A549 cells [93].

Having an understanding of the apoptotic mechanism induced by the lead compound and the advantages and the limitations of the compound structure on different cancer types will increase the lead compounds chances of developing into a recognized targeted cancer therapy. Furthermore, if it were only a matter of bioactivity, or having a unique pharmacological profile, hundreds of sponge-derived compounds would enter drug development in the pharmaceutical industry. However, other factors besides bioactivity such as a cost-effective source of large-scale supply, and drug ability will determine whether the lead compound enters the drug development pipeline [20]. Sponge-derived compounds that are currently in clinical trials were all found to be amenable to cost-effective chemical synthesis, thus solving the supply question early-on in the development continuum [124–126]. Thus far, the chemical synthesis of the apoptosis-inducing lead compounds listed in Table 1 include Renieramycin M [127], Naamidine A [128], Psammaplysenes A and B [129], 13*E*,17*E*-globostellatic acid X methyl ester [130], Pachastrissamine [131,132], Spongistatin 1 [133,134] and Irciniastatin A [135,136].

## 5. Conclusions

This review highlights recently confirmed apoptosis-inducing compounds isolated from marine sponge that have the potential to be developed into targeted cancer therapies. There are numerous cytotoxic metabolites that have been isolated from marine sponge but have not been screened for apoptosis activity. Moreover, the effects of the apoptosis-inducing compounds on the apoptotic signaling pathways have been summarized, even though most of these compounds have not yet been studied in depth for their mechanisms of action. Further research needs to be focused on clarifying the apoptotic pathways induced by the potential anticancer drugs to help singling out those that are the most promising for development of new anticancer drugs.

## Figures and Tables

**Table 1:** Recently confirmed apoptosis-inducing compounds isolated from marine sponges.

Compound	Type	Sponge	Cell line	Reference
(**1**) 2,3-dihydro-2,3-dioxoaaptamine	alkaloid	*Aaptos* sp.	THP-1	[26] (abstract)
(**2**) 3-(methylamino)demethyl (oxy)aaptamine	alkaloid	*Aaptos* sp.	THP-1	[26] (abstract)
(**3**) 6-(*N*-morpholinyl)-4,5-dihydro-5-oxo-demethyl (oxy)aaptamine	alkaloid	*Aaptos* sp.	THP-1	[26] (abstract)
(**4**) kuanoniamine A	alkaloid	*Oceanapia sagittaria*	MCF-7	[27]
(**5**) kuanoniamine C	alkaloid	*Oceanapia sagittaria*	MCF-7	[27]
(**6**) Makaluvic acid C	alkaloid	*Strongylodesma aliwaliensis*	WHC01 WHC06 KYSE30	[28]
(**7**) Monanchocidin	alkaloid	*Monanchora pulchra*	THP-1 HeLa	[29]
(**8**) *N*-1-β-d-ribofuranosyl damirone C	alkaloid	*Strongylodesma aliwaliensis*	WHC01 WHC06 KYSE30	[28]
(**9**) *N*-1-β-d-ribofuranosyl makalu-vamine I	alkaloid	*Strongylodesma aliwaliensis*	WHC01 WHC06 KYSE30	[28]
(**10**) *N*-1-β-d-ribofuranosyl makaluvic acid C	alkaloid	*Strongylodesma aliwaliensis*	WHC01 WHC06 KYSE30	[28]
(**11**) Naamidine A	alkaloid	*Leucetta chagosensis*	A-431	[30]
(**12**) Psammaplysene A	alkaloid	*Psammaplysilla*	Ishikawa ECC1	[31]
(**13**) Renieramycin M	alkaloid	*Xestospongia* sp.	NSCLC	[32]
(**14**) Bastadin 6	alkaloid	*lanthella* sp.	HUVEC	[33] (abstract)
(**15**) (*Z*)-stellettic acid C	lipid	*Stelletta* sp.	U937	[34] (abstract)
(**16**) 5-alpha,6-alpha-*epoxy*petrosterol	lipid	*Lanthella* sp.	HL-60	[35]
(**17**) Callyspongidiol	lipid	*Callyspongia* sp.	HL-60	[36] (abstract)
(**18**) Jaspine B	lipid	*Jaspis* sp.	SK-Mel28	[37]
(**19**) Petrosterol	lipid	*Lanthella* sp.	HL-60	[35]
(**20**) Petrosterol-3,6-dione	lipid	*Lanthella* sp.	HL-60	[35]
(**21**) Rhizochalin	lipid	*Rhizochalina incrustata*	THP-1 HeLa SNU-C4	[38]
(**22**) Candidaspongiolide	macrolide	*Candidaspongia* sp.	U251 HCT116	[39]
(**23**) Irciniastatin A	macrolide	*Ircinia ramose*	Jurkat T	[40]
(**24**) Latrunculins A and B	macrolide	*Negombata magnifica*	MKN45 NUGC-4	[41] (abstract)
(**25**) Salarin C	macrolide	*Fascaplysinopsis* sp.	K562	[42]
(**26**) Spongistatin 1	macrolide	*Spongia*	Jurkat T	[43]
(**27**) 13*E*,17*E*-globostellatic acid X methyl ester	terpenoid	*Rhabdastrella globostellata*	HUVEC	[44]
(**28**) 3-*epi*-sodwanone K 3-acetate	terpenoid	*Axinella* sp.	T47D	[45]
(**29**) Geoditin A	terpenoid	*Geodia japonica*	HL-60 HT29	[46] [47]
(**30**) Geoditin B	terpenoid	*Geodia japonica*	HL-60	[46]
(**31**) Heteronemin	terpenoid	*Hyrtios* sp.	K562 Jurkat T	[48]
(**32**) Ilimaquinone	terpenoid	*Hippospongia metachromia*	PC3	[49]
(**33**) Jaspolide B	terpenoid	*Jaspis* sp.	Bel-7402 HepG2	[50]
(**34**) Rhabdastrellic acid-A	terpenoid	*Rhabdastrella globostellata*	HL-60	[51]
(**35**) Sipholenol A	terpenoid	*Callyspongia siphonella*	KB-3-1 KB-C2 KB-V1	[52]
(**36**) Smenospongine	terpenoid	*Dactylospongia elegans*	K56	[53]
(**37**) Sodwanone V	terpenoid	*Axinella* sp.	MDA-MB-231	[45]
(**38**) Stellettin A	terpenoid	*Geodia japonica*	HL-60	[46]
(**39**) Stellettin B	terpenoid	*Geodia japonica*	HL-60	[46]
